# Isolation and characterization of bovine coronavirus variants with mutations in the hemagglutinin-esterase gene in dairy calves in China

**DOI:** 10.1186/s12917-025-04538-w

**Published:** 2025-02-24

**Authors:** Long Zhao, Dong Wang, Huihua Jiang, Qingyun Gu, Haihui Gao, Liang Zhang, Wenhui Liu, Shengqing Li, Xiaodong Kang, Kangkang Guo

**Affiliations:** 1 College of Veterinary Medicine, Northwest A&F University, No. 22 Xinong Road, Yangling, Shaanxi 712100 China; 2 Tibet Vocational Technical College, Lhasa, Tibet 850030 China; 3 Qinghai Academy of Animal Science and Veterinary Medicine, Qinghai University, No. 1 Weier Road, Xining, Qinghai 810003 China; 4 Institute of Animal Science, Ningxia Academy of Agricultural and Forestry Sciences, No. 590, Huanghe East Road, Yinchuan, Ningxia 750002 China

**Keywords:** Bovine coronavirus, Hemagglutinin-esterase, Variant, Isolation, Characteristics

## Abstract

**Background:**

Bovine coronavirus (BCoV) is a causative agent of enteric and respiratory diseases in cattle and is responsible for severe economic losses. Recently, a novel BCoV variant with 12-nucleotide deletion or insertion in the hemagglutinin-esterase (HE) receptor-binding domain (RBD) has emerged. However, the biological consequences of these deletions/insertions and the prevalence of these variants remain unknown. Here, 47 diarrheal and 47 nasal swab samples were collected from five cattle farms in various Ningxia, China regions to detect and isolate BCoV.

**Results:**

Eleven complete HE genes and eight complete S genes were amplified from 34 BCoV-positive samples using RT-PCR. Eight BCoV strains were successfully isolated using HRT-18 cells, and four underwent genome sequencing. Three HE genes contained a 12-nucleotide insertion in the RBD, and a single HE gene contained a novel 12-nucleotide deletion. Phylogenetic analysis of genomes revealed that these HE-deletion/insertion variants do not share a common most recent ancestor with those reported from the US. Molecular docking results showed that the insertion of four additional amino acids between F211 and L212 increased the affinity of HE protein to O-acetylated sialic acid, which may be favorable for virion-particle attachment. Growth kinetics suggest that the HE-deletion variant had a non-cytopathic effect and lower virus titer.

**Conclusions:**

These findings suggest that BCoV HE deleted/inserted variants are prevalent in cattle and exhibit various biological characteristics. We should be alert to these HE-variants with insertions or deletions in the RBD, which may increase the possibility of interspecies transmission.

**Supplementary Information:**

The online version contains supplementary material available at 10.1186/s12917-025-04538-w.

## Background

Bovine coronavirus (BCoV) is widespread in cattle and wild ruminant populations; it causes diarrhea, winter dysentery, and the bovine respiratory disease complex, resulting in considerable economic losses [1, 2]. BCoV is a single-stranded positive-sense RNA virus with a lipid envelope that belongs to the species *Betacoronavirus 1* (subgenus *Embecovirus*) of the *Betacoronavirus* genus with equine coronavirus, wild ruminant coronaviruses, porcine hemagglutinating encephalomyelitis virus, human enteric coronavirus 44, human coronavirus OC43 (HCoV-OC43), and canine respiratory coronavirus (ICTV: https://talk.ictvonline.org/). BCoV showed tissue tropism in the respiratory and gastrointestinal tract [3]. Although some studies identified mutations that may be associated with intestinal and respiratory phenotypes [4–6], these findings have not been confirmed in experimental studies [7]. In addition to cattle and wild ruminant populations, BCoV-like coronaviruses have been detected in humans [8]. HCoV-OC43 emerged from a BCoV spillover [9], demonstrating the possibility of zoonosis.

BCoV possesses an RNA genome of approximately 31 kb. ORF1a/b encodes the replicase polyproteins pp1a and pp1b, respectively, occupying two-thirds of the 5' end of the genome. The 3’ end encodes five major structural proteins: spike (S), hemagglutinin-esterase (HE), integral membrane (M), envelope (E), and nucleocapsid (N) [10]. The S protein mediates pathogenesis, viral entry, and host cell attachment [11, 12]. It consists of S1 and S2 subunits [11, 13]. S1 mediates the binding of viruses to host cell receptors, possesses hemagglutinin activity, and induces neutralizing antibody production [11]. S2 mediates the fusion process of the virus and host cell membrane [11]. The HE protein contains an esterase, lectin, and membrane-proximal domain [14]. At the initial stage of viral infection, the HE protein acts as a secondary attachment protein to help the virus adsorb [15]. Due to the presence of the esterase domain, the lectin domain of HE can reversibly bind to receptors on the cell surface, which is critical for viral infection and entry [16, 17].

SARS-CoV-2 began to infect humans and became a global pandemic, raising the specter of potential future outbreaks of zoonotic coronavirus disease in humans [9]. Recently, a novel BCoV HE variant with a 12-nucleotide deletion or insertion in the HE receptor-binding domain (RBD) has emerged [18, 19]. The mutations in the RBD increase the possibility of BCoV interspecies transmission [18]. HCoV-OC43 HE loses its receptor-binding function through mutation accumulation and adapts to replication in the human respiratory tract [20]. This indicates that mutations in the HE protein may similarly modify the tissue tropism of BCoV, potentially resulting in more severe infections in humans or animals. However, the biological consequences of these deletions/insertions and the prevalence of these BCoV variants remain unknown. Therefore, this study aims to characterize the biological properties of novel BCoV HE-deletion/insertion variants and assess their effect on the binding affinity of the HE protein to its receptor.

## Results

### BCoV detection and amplification of genes

Among the 47 diarrheal and 47 nasal swab samples, 10 (21.28%) and 24 (51.06%), respectively, were BCoV-positive as detected by RT-PCR (see additional file 1). The positivity rate for BCoV in nasal swabs was significantly higher than that of diarrheal samples (*P* < 0.01). BCoV was detected in all five cattle farms. Eleven complete HE (GenBank accession No. OP762501–OP762502, OP762507–OP762511, and OP866726–OP866729) and eight complete S genes (GenBank accession No. OP762512–OP762515 and OP866726–OP866729) were successfully amplified from 34 BCoV-positive samples using RT-PCR.

### BCoV isolation and identification

**Fig. 1:**
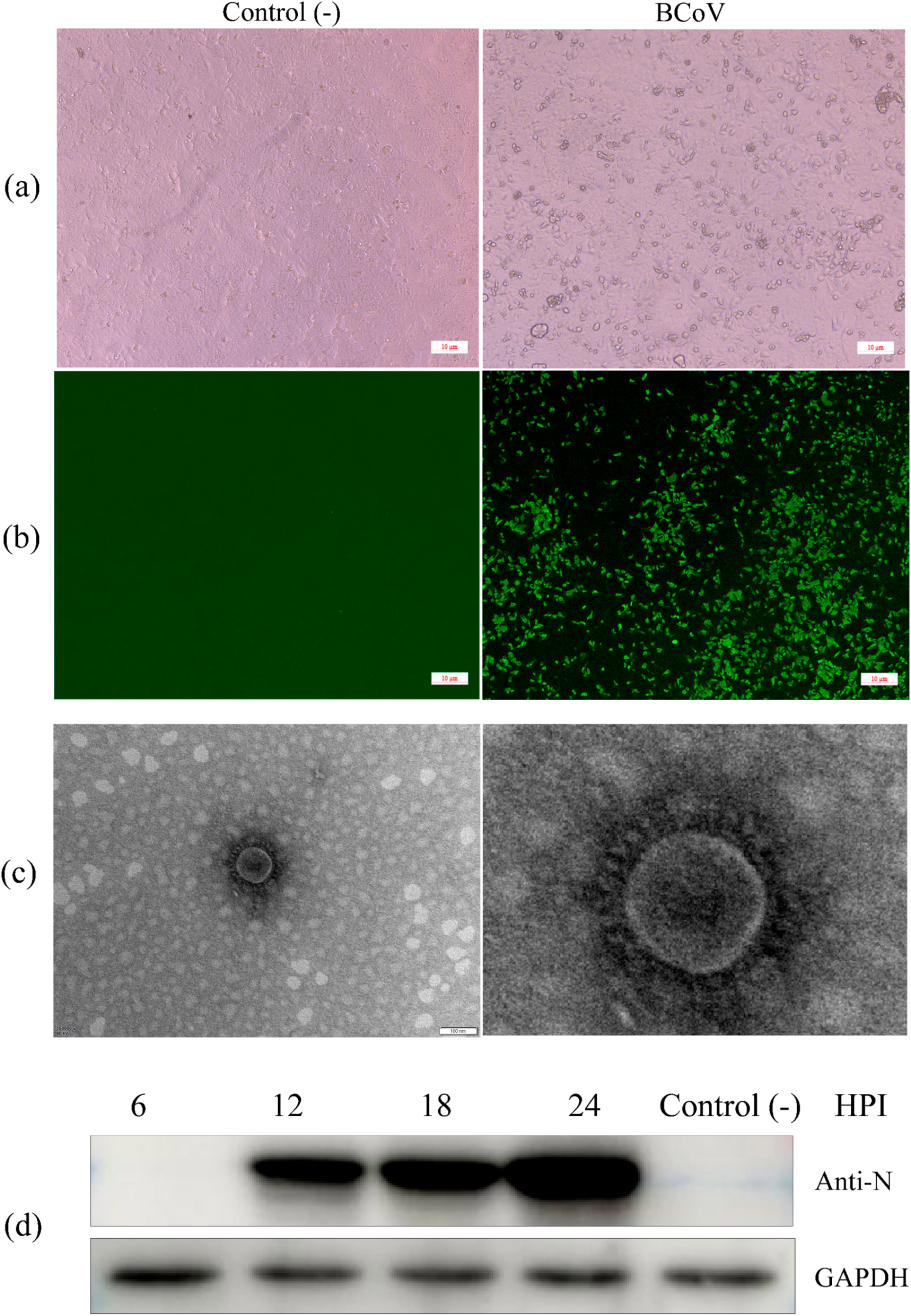
Isolation and identification of BCoV. (**a**) HRT-18 cell infected with BCoV showing cytopathic effects (CPE) at 72 h, such as shrinkage, rounding, and nonadherence. (**b**) IFA of BCoV-infected HRT-18 cells using the anti-N antibody. (**C**) TEM images of BCoV. (**d**) The viral protein N was detected with western blotting using an anti-N Polyclonal antibody at 6, 12, 18, and 24 h post-infection. Full-length blots are presented in additional file 9. The black box is the cropped area

Thirty-four BCoV-positive samples were inoculated into HRT-18 cell cultures; seven samples displayed evident cytopathic effect (CPE), characterized by round shrinkage and shedding 60–72 h in generation three, with stable CPE observed approximately 72 h after generation 5 (Fig. 1a). The seven viral isolates were plaque-purified three times at passage six; two were HE-insertion variants(B298-HE428 and B299-HE428). The remaining strains were HE non-variant. RT-PCR identified one virus isolate as only BCoV-positive and did not show evident CPE; this strain was identified as an HE-deletion variant (B277b-HE420). Specific green fluorescence could be observed by indirect immunofluorescence assay (IFA) in the eight isolates (Fig. 1b), and the virus particles with crown-like morphology on the surface could be observed by electron microscopy (Fig. 1c). Western blot results showed that BCoV-N protein was expressed at 12 h post-infection (HPI), and the expression level increased with infection time (Fig. 1d), suggesting that the BCoV strain was successfully isolated. We successfully isolated two and six BCoV strains from ten positive feces and 24 nasal swabs respectively. The titers of the isolates are listed in Table 1.

**Table 1:** Information on BCoV strains isolated in China in this study

No	Strain Name	Sample Type	Titer Determined (TCID_50_/mL)	HE gene	GenBank Accession for complete genome
1	B277b-HE420	swab	10^− 7.51^	_208_NGKF_211_ deletion	OP866726
2	B298-HE428	swab	10^− 9.14^	_212_KATV_215_ insertion	OP866728
3	B277a-HE424	swab	10^− 9.32^	/	OP866729
4	F226-HE424	feces	10^− 5.50^	/	OP866727
5	B299	swab	10^− 3.77^	_212_KATV_215_ insertion	/
6	B236	swab	10^− 7.4^	/	/
7	B226	swab	10^− 4.76^	/	/
8	F327	feces	10^− 7.51^	/	/

### Genomic characterization of isolates

The complete genomes of the HE-insertion variant (B298-HE428), HE-deletion variant (B277b-HE420), HE non-variant (B277a-HE424), and enteric isolate (F226-HE424) were sequenced and submitted to GenBank with accession number OP866726–OP866729 (Table 1). The four linear genomes were 31 023–31,044 nt in length with a G + C content of 36.97–37.07%. Specifically, they possessed standard bovine coronavirus genome organization: ORF1a and ORF1b encode nonstructural replicase polyproteins, encoding five major structural proteins: S, HE, M, E, and N. Sequence comparisons revealed that all four genomes share 98.9% nt identity and 97.8–99.5% nucleotide identity with all 212 BCoV genomes available in GenBank. Additional file 2 displays the nucleotide identity and amino acid (aa) identity of individual genes of B298 compared with three other genomes and four representative genomes. Compared with the reference enteric strain Mebus, the most significant variation in the genome was the presence of four aa deletions or insertions in the HE gene, while none of the ORF1a/b, S, M, E, and N genes were deletions or insertions. The S and HE genes of the four isolates were consistent with the amplified sequences of the corresponding clinical samples.

Compared with other strains, strain B298-HE428 had four unique aa variants (S75N, G468C, L2765F, W3646C) and two unique aa variants (L268I and P2452S) in the ORF1a gene and ORF1b gene, respectively. Strains B277a-HE424 and F226-HE424 had three identical aa variants (K1665R, T2743I, Q2820R) in the ORF1a gene, and strain B277b-HE420 had one unique aa variant (R3197K) in the ORF1a gene. No unique aa mutations existed in the M, E, and N genes.

**Fig. 2:**
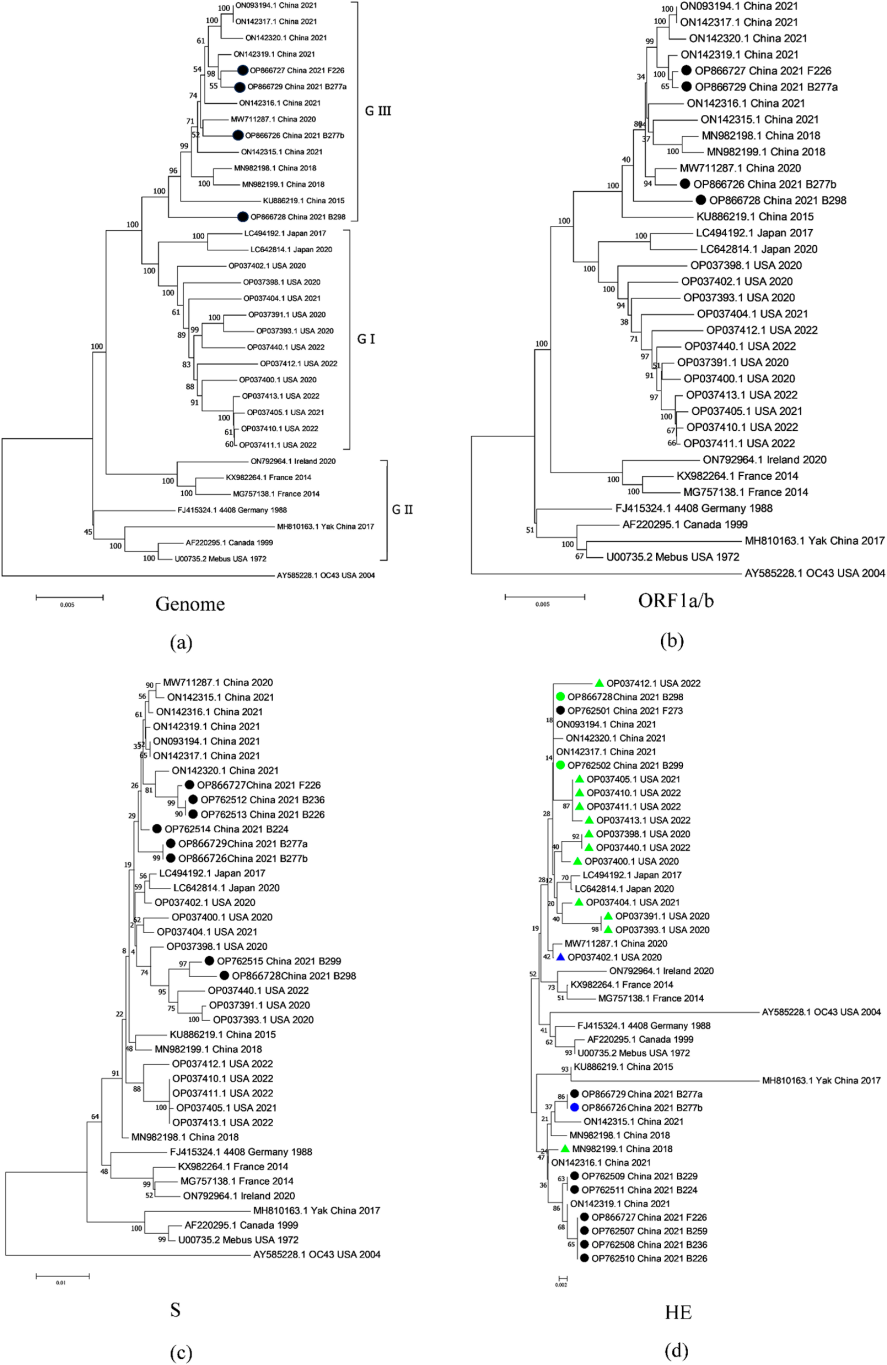
Maximum-likelihood analysis in combination with 1,000 bootstrap replicates was used to derive a phylogenetic tree based on the complete genomes (**a**), ORF1a/b genes (**b**), S (**c**), and HE (**d**) protein sequences. The sequences were aligned and clustered by Clustal W in MEGA 7.0 software. Circles represent the BCoV strains from this study and the HE-insertion variants were marked with a green triangle and the HE-deletion variants were marked with a blue triangle

Phylogenetic analysis of the complete genome sequences revealed that, except for the Chinese yak strain, the other Chinese BCoV strains (including the strains in this study) clustered into a large independent branch, showed a unique evolution, and belonged to genotype GIII (Fig. 2a). The HE-deletion/insertion variants in this study did not cluster phylogenetically with the HE-deletion/insertion variants from the US, suggesting that the Chinese and US deletion/insertion variants did not share a recent common ancestor (Fig. 2a). The respiratory (B277a-HE424) and enteric (F226-HE424) isolates did not cluster according to disease presentation but instead, clustered in the same branch according to geographic location, consistent with the phylogenetic analysis of ORF1ab gene (Fig. 2b).

Phylogenetic analysis of the M and N genes revealed that, except for strain B298-HE428, which had the closest genetic relationship with the US strain OP037391.1, the other strains were clustered with the Chinese strain (see additional file 3). Phylogenetic analysis based on the E genes showed that strain B277a-HE424 was clustered with the U.S. strain AY585228.1 (see additional file 3). The strain F226-HE424 was clustered with the Chinese yak strain MH810163.1. The strain B277b-HE420, B298-HE428, and U.S. strain OP37405.1 were clustered together.

### Molecular characterization of the S genes

At 4,092 bp each, the eight S genes encode a 1,363 aa protein. A phylogenetic tree based on complete S gene sequences showed that the S genes of the eight strains clustered in three branches of the genetic tree, indicating genetic diversity (Fig. 2c). Similar to the genome, BCoV isolates could not be phylogenetically differentiated based on their clinical sources (respiratory or enteric).

Unlike other BCoV S genes, the enteric strain F226-HE424 (GenBank accession No. OP866727) and respiratory strains B236 and B226 (GenBank accession No. OP762512 and OP762513), located in an independent branch (Fig. 2c), each had an identical aa variant (N338S) in the S1 subunit. Strain F226-HE424 had a unique aa variant (E858G) in the S2 subunit. The strain B298-HE428 (GenBank accession No. OP866728) had a unique aa variant (F468L) in its S1 subunit. No frameshifts, deletions, or insertions were observed in the S gene sequences of any strain.

### Molecular characterization of the HE genes

The eleven HE genes were 1,263–1,287 bp long and encoded a 420–428 aa protein. A phylogenetic tree based on the complete HE gene sequences showed that the HE genes of the eleven strains clustered in four different branches of the genetic tree (Fig. 2d), indicating genetic diversity. The respiratory and enteric strains clustered according to geographical origin and date of isolation rather than disease presentation.

Compared to other BCoV HE genes, the three strains from the two different farms in this study (GenBank accession No. OP762501, OP762502, and OP866728) contained an identical insertion of 12 nucleotides (AAGGCTACTGTT), resulting in four additional aa (KATV) between F211 and L212 in the R3-loop. The strain B277b-HE420 (GenBank accession No. OP866726) had a deletion of 12 nucleotides (AATGGCAAGTTT) in the R3 loop of the HE gene, resulting in the deletion of aa 208NGKF211 (Fig. 2a). Notably, the HE-deletion variant in this study did not cluster phylogenetically with the HE-deletion variant from the U.S. (GenBank accession No. OP037402), suggesting that the Chinese and US deletion variants do not share a common most recent ancestor (Fig. 2d).

### Molecular docking

**Fig. 3:**
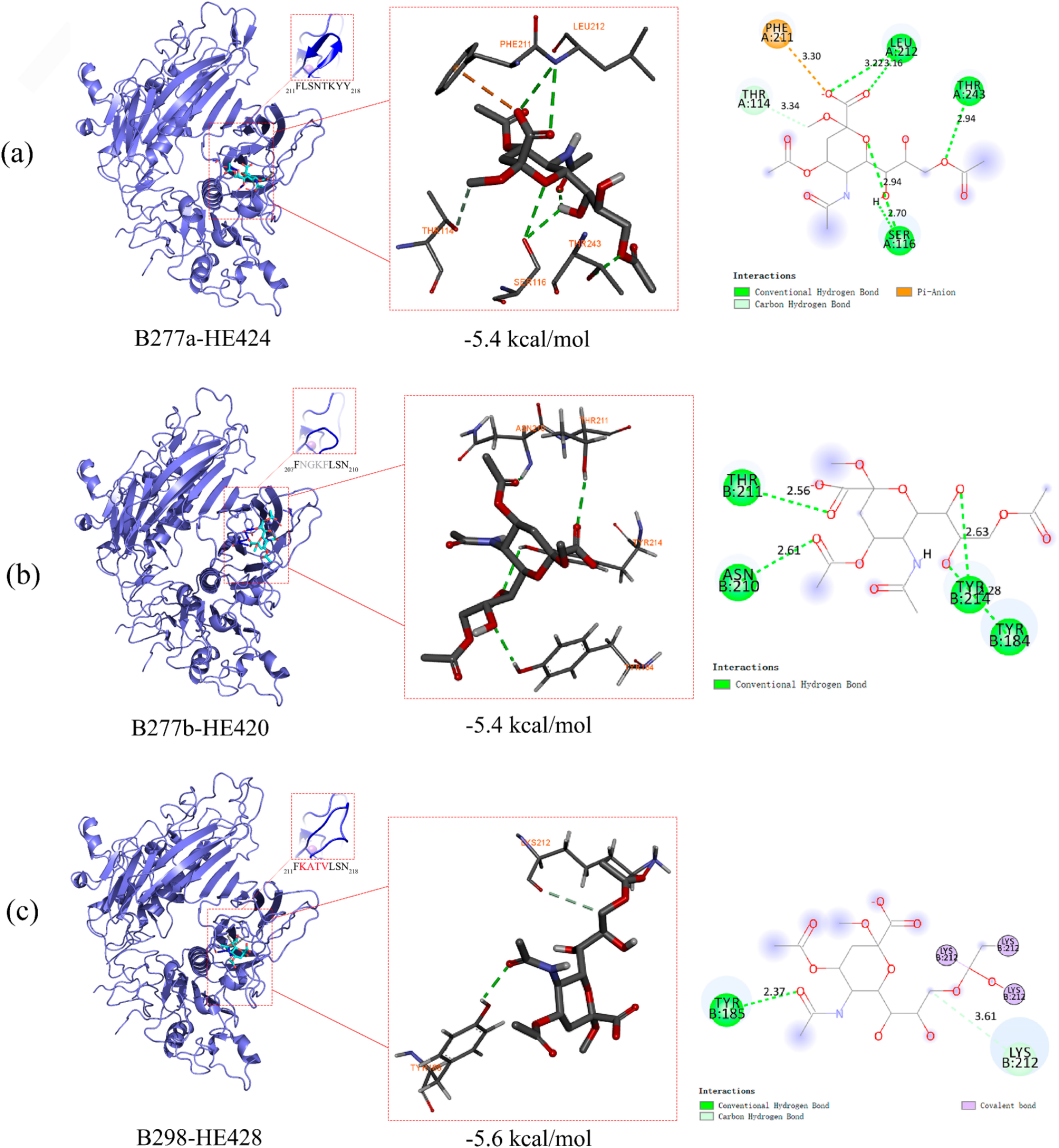
The molecular docking models of HE protein and O-acetylated sialic. (**a**) The molecular docking structure of B277a-HE424 and O-acetylated sialic. The docking energy is -5.4 kcal/mol. (**b**) The molecular docking structure of B277b-HE420 and O-acetylated sialic. The docking energy is -5.4 kcal/mol. (**c**) The molecular docking structure of B298-HE428 and O-acetylated sialic. The docking energy is -5.6 kcal/mol. The boxed portions of the structures indicate the different conformations of the R3-loop. The dotted green lines represent hydrogen bonds. The light green lines represent carbon-hydrogen bonds. The pink lines represent hydrophobic forces. The purple circles represent aa residues where covalent forces occur

Three-dimensional models constructed based on the crystal structure of bovine coronavirus HE (SMTL ID: 3cl4.1) showed that the insertion of four additional aa (KATV) between F211 and L212 and the deletion of aa 208NGKF211 disrupted the beta-sheet secondary structure that formed the RBD (Fig. 3). Molecular docking results showed that O-acetylated sialic could spontaneously bind with strains B277a-HE424, B277b-HE420, and B298-HE424 in the RBD. If the binding energy of the ligand to the target protein is less than 0, the ligand and the receptor protein can spontaneously bind. Binding energy between an active compound and a target protein receptor is less than − 5 kcal/mol is considered good binding activity. The binding energies of O-acetylated sialic and strains B277a-HE424, B277b-HE420, and B298-HE424 were − 5.4 kcal/mol, -5.4 kcal/mol, and − 5.6 kcal/mol, respectively. Being lower than − 5 kcal/mol suggests that O-acetylated sialic and B277a-HE424, B277b-HE420, and B298-HE424 have a strong affinity. Compared with the B277a-HE424, due to the insertion of four additional aa (KATV) between F211 and L212, the affinity of strain B298-HE424 to ligand O-acetylated sialic elevated (-5.6 kcal/mol < -5.4 kcal/mol) (Fig. 3c), while the deletion of aa 208NGKF211 in strain B277b-HE420 did not change the binding energy (Fig. 3b).

### One-step growth curve

**Fig. 4:**
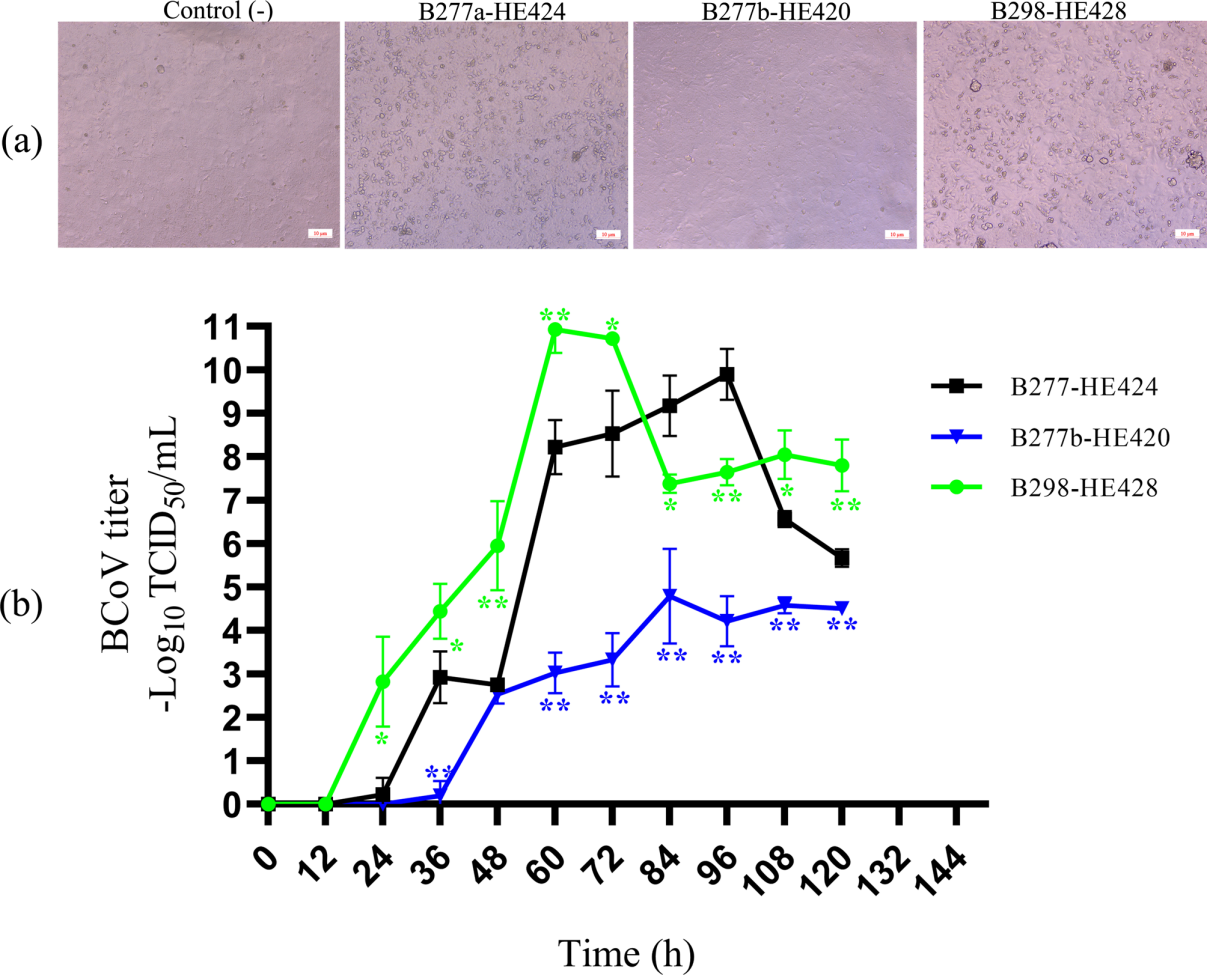
One-step growth curve analysis. (**a**) HRT-18 cell were infected with the HE-insertion variant (B298-HE428) and HE non-variant (B277a-HE424) showing CPE at 72 h, while the HE-deletion variant (B277b-HE420) showed non-CPE. (**b**) Growth kinetics of the HE-insertion variant (B298-HE428), HE-deletion variant (B277b-HE420), and HE non-variant (B277a-HE424) were depicted by one-step growth curve. The HRT-18 cells were infected with 1 MOI virus. Cell culture was harvested at 12 h interval and TCID_50_ was calculated. The mean values with standard deviations of three independent experiments were calculated. *, *P* < 0.05; **, *P* < 0.01, compared with the HE non-variant (B277a-HE424)

The one-step growth curve revealed that the HE-insertion variant (B298-HE428) and HE non-variant (B277a-HE424) displayed evident CPE, characterized by round shrinkage and shedding at 72 h, while the HE-deletion variant (B277b-HE420) did not show CPE (Fig. 4a). The HE non-variant (B277a-HE424) exhibited lower virus titers at 24, 36, 48, 60, 72, 108, and 120 HPI (*P* < 0.05) than the HE-insertion variant (B298-HE428) but relatively higher titers than the HE-deletion variant (B277b-HE420) at 36, 60, 72, 84, 96, 108, and 120 HPI (*P* < 0.01) (Fig. 4b). These findings suggest that the HE-deletion variant had a non-cytopathic effect and lower virus titer than the HE non-variant, whereas the HE-insertion variant showed a cytopathic effect and higher virus titer.

### Recombination analysis

**Fig. 5:**
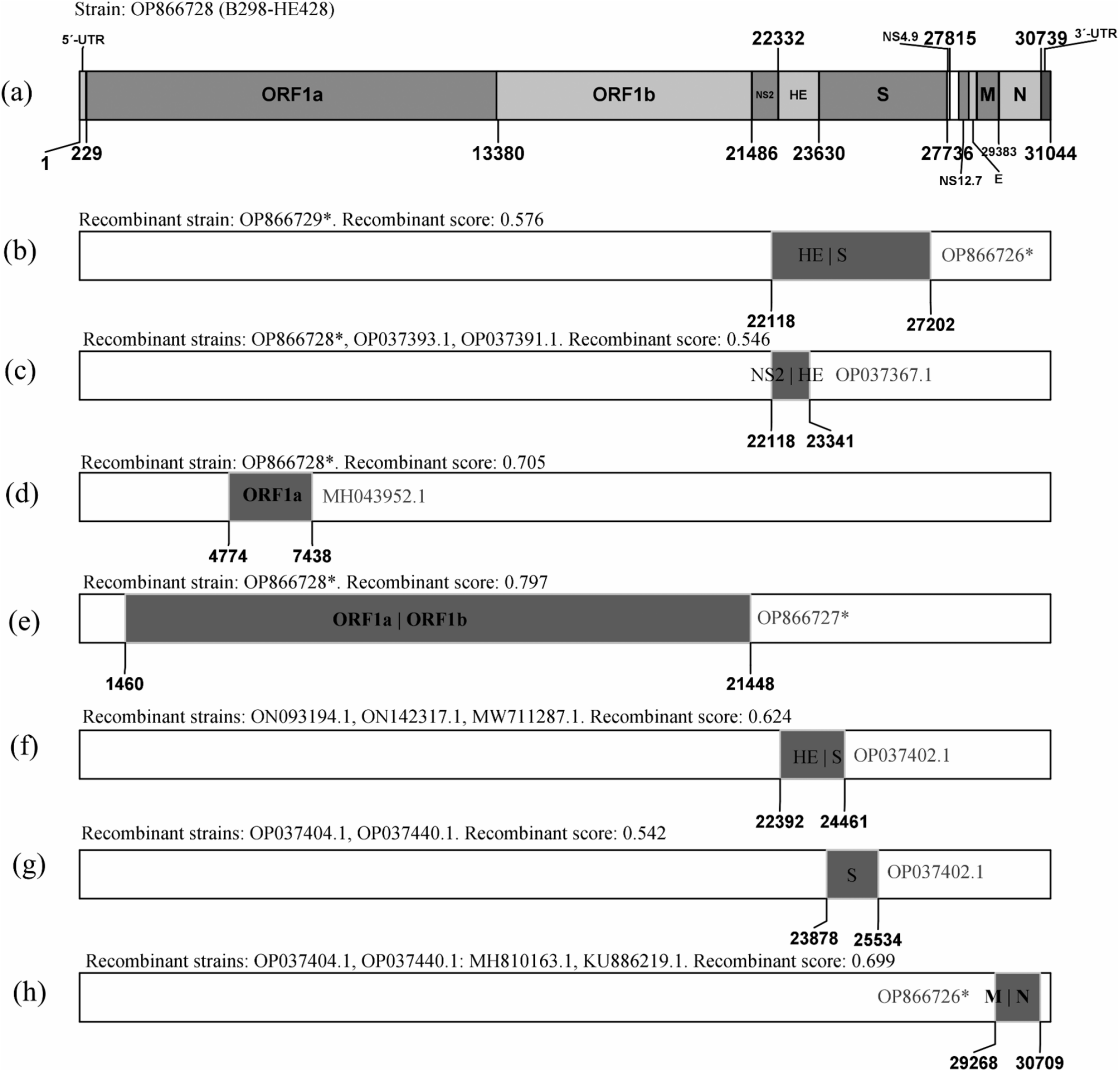
Schematic diagram showing the genomic organization and potential recombination events of BCoV isolates. (**a**) Genomic organization of the HE-insertion variant (B298-HE428) from this study (GenBank accession No. OP866728). The numbers represent the location in the genome. (**b**)-(**h**) Schematic diagram showing the potential recombination events among different BCoV isolates. The genomic locations and breakpoints of the potential recombining segments are schematically indicated with grey frames. *, the new isolates in this study

To determine the origin of the HE-variants in the US and China, recombination profile analysis was performed for 216 non-redundant BCoV genomes, including the four new isolates. We identified seven recombination events potentially occurring in BCoV, and most events (4/7) contained recombination breakpoints located in the S and HE genes (Fig. 5), based on the RDP 4.0 and SimPlot 3.5.1 (see additional file 4). The HE-deletion variant (GenBank accession No. OP866726) was predicted to be a possible minor parental strain of the recombination event in the HE-S region (Fig. 5b).

A recombination event was predicted in the NS2-HE region of the HE-insertion variant (GenBank accession No. OP866728) in this study (recombinant score = 0.546) (Fig. 5c). The putative major parental strain was BCoV1/2021/CHN, identified in China (GenBank accession No. ON142315.1), and the possible minor parental strain was MARC/2014/01/R (GenBank accession no. OP037367.1), from the US. HE-insertion variants from the US (GenBank accession No. OP037393.1 and OP037391.1) were also identified as recombinant strains, and the recombination events were the same as those in this study.

## Discussion

BCoV causing respiratory and enteric diseases are widespread in cattle farms worldwide and cause severe economic losses [21–23]. In the present study, the detection rate of BCoV in nasal swabs was significantly higher than in feces, suggesting that BCoV is also a significant respiratory pathogen [24], and respiratory BCoV should be included in detecting respiratory pathogens. Co-infection or virus shedding of the respiratory and intestinal tracts may explain the extensive spread of BCoV [25, 26]. We successfully isolated eight BCoV strains from the samples. Interestingly, BCoV is more likely to be isolated and cultured from nasal swab samples. This finding may be due to the complex composition of feces, which is not conducive to the culture of viruses, suggesting that we may be more likely to succeed in selecting nasal swab samples for BCoV isolation and culture.

Sequence alignment analysis showed that the aa mutations of the strains obtained in this study mainly existed in ORF1 a, ORF1 b, HE, and S genes, and the deletion or insertion of amino acids mainly existed in the HE gene. The S protein is involved in antigenic diversity, host specificity, immunogenicity, and receptor recognition [11]. Mutations in this protein are associated with alterations in tissue tropism, host range, viral antigenicity, and pathogenicity [13, 27]. Unlike the other S genes available in GenBank, three of eight of our sequences clustered on an independent branch of the phylogenetic tree, and each had an identical aa variant (N338S) in the S1 subunit. Strain B298 had a unique aa mutation (F468L) in the S1 domain, and strain F226 had an aa mutation (E858G) in the S2 subunit. Previous studies showed that point mutations in the S1 subunit of the S protein may reduce the binding of the virus to the cell receptor, avoiding the binding of BCoV to neutralizing antibodies [28]. The mutation of the S2 subunit of mouse hepatitis virus (MHV) affects the ability of the S1 subunit to bind to receptors and mediate host range expansion [29, 30]. Therefore, unique aa mutations in these regions may affect the virus’s ability to bind to antibodies. Future efforts in vaccine development or the design of therapeutic antibodies should prioritize the assessment of the immunogenicity and antigenicity of these variant strains.

Receptor-binding region of HE protein consists of six surface loops, among which five are grafted onto the beta‐sandwich core of the lectin domain, named the R1‐loop, R2‐loop, R3‐loop, R4‐loop, and the RBS‐hairpin [20]. The R3-loop is composed of 13 aa (aa 207–219) in HE [14]. The HE-Phe211Ala substitution abolishes HE-mediated virion attachment to O-acetylated sialic acid determinants and reduces overall virion binding avidity [14]. L212, S213, and N214 are essential for receptor-ligand interactions [14]. We found three isolates with identical insertions of four aa (KATV) between F211 and L212 in the R3-loop. The HE-deletion variant contained a novel deletion of aa 208NGKF211 in the R3-loop; both altered the spatial conformation of the receptor binding site. This finding suggests that the 4-aa insertion or 4-aa deletion in the R3-loop may affect HE receptor binding. Molecular docking results confirmed our hypothesis that the insertion of four additional aa (KATV) between F211 and L212 increased the affinity of strain B298-HE428 to O-acetylated sialic acid, which may be more favorable for virion-particle attachment. However, these predictions are based on bioinformatics approaches and have not yet been experimentally validated. Therefore, the development of a reverse genetic operating system for BCoV will be a key focus of our future work.

For betacoronavirus-1 members, S and HE are functionally interdependent and coevolve to optimize the balance between attachment and release [31]. Amino acid deletions/insertions in HE proteins may alter this balance. Future research will prioritize providing more direct evidence or experimental analysis to confirm changes in this balance and link mutations to alterations in host range or tissue tropism. Reverse genetic systems have been established for several animal coronaviruses, including transmissible gastroenteritis virus, porcine epidemic diarrhea virus, infectious bronchitis virus, feline coronavirus, and MHV, yet BCoV remains one of the few animal coronaviruses without such a system [32]. The large size of the coronavirus genome, coupled with the toxicity and instability of the cDNA sequence in bacterial systems, are likely the primary factors hindering the development of reverse genetics systems for these viruses. The SARS-CoV-2 pandemic, however, has driven the development of three novel reverse genetics approaches for coronaviruses: yeast-based transformation-associated recombination cloning, infectious subgenomic amplicons, and circular polymerase extension reaction [32]. These methods hold considerable promise for future application in the establishment of a reverse genetic system for BCoV. Analysis of growth properties showed that the HE-deletion variant had a non-CPE and lower virus titer than the HE non-variant, whereas the HE-insertion variant showed a cytopathic effect and higher virus titer. The non-CPE of the HE-deletion variant may be conducive to establishing a recessive infection, and a low level of replication in cells can avoid immune clearance [33]. Notably, BCoV infectivity varies across different cell types, including HRT-18 cells and various bovine cell lines [18]. HRT-18 cells are commonly used for BCoV isolation due to their high susceptibility and robust viral production. Interestingly, BCoV did not infect bovine ileal epithelial cells but was able to infect bovine enteroids (organoids derived from the intestine) [34, 35]. These differences may be attributed to variations in the distribution of sialic acid receptors on the surface of different cell types. The biology of these variant strains should be further investigated using a broader range of bovine cell models in future studies.

In 2020, Abi et al. detected a HE-insertion variant in dairy cattle in Liaoning, China [19]. Here, the HE-insertion variant was detected again in dairy cows in Ningxia (approximately 1800 km apart), indicating that the HE-insertion variant is endemic in China (see additional file 5). Recently, Workman et al. reported a HE-insertion variant and a new HE-deletion variant in the US [18]. This HE-deletion variant was also identified in China in the present study. To our knowledge, ours is the first report of the HE-deletion variant in China. These HE-deletion/insertion variants may be an emerging pattern in BCoV evolution [19]. Although phylogenetic analysis of genomes revealed that the HE-deletion/insertion variants in this study did not share a recent common ancestor with those reported in the US, incorporating global surveillance data in future studies could provide more comprehensive insights into BCoV evolution, helping to identify broader patterns and potential regional or temporal factors influencing viral diversity. Phylogenetic analysis revealed that the respiratory and enteric isolates did not cluster according to disease presentation but instead clustered in the same branch according to geographic location suggests that genetic variation may not always correlate with clinical outcomes. Previous studies have demonstrated that following experimental infection with a single BCoV strain, both the gastrointestinal and respiratory tracts exhibit severe pathological lesions, with viral antigens detected in both tissues [3, 36]. Therefore, the respiratory and intestinal isolates are likely derived from the same viral strain, as no distinct genetic or antigenic markers specific to either the respiratory or intestinal isolates were identified, consistent with previous findings [18, 37, 38]. Recombination analysis showed that the HE-insertion variant was obtained in our study, and there were recombination breakpoints in the US in the NS2-HE region. The HE-deletion variant in this study was also predicted to be a possible minor parental strain for recombination events located in the HE-S region. In 2019, a novel BCoV strain carrying the recombinant HE gene was detected in dairy cattle in China [39]. The recombination of the HE gene facilitates virus evolution and can result in the emergence of new pathotypes and the expansion of host ranges and tissue tropism change [15, 39–41]. Future studies should experimentally infect mice, pigs, goats, and other animals with these variant strains to assess interspecies transmission risks. We speculate that the HE-insertion/deletion variant may have been obtained by recombination with the ancestor virus with a 12-nt insertion/deletion. However, no 12-nt insertion or deletion was found in the HE gene of the predicted parent strain, which may be because the putative common ancestor of these HE-variants was not submitted to GenBank [18]. Future extensive genomic surveillance and sequencing efforts are needed to address this gap. The study’s geographic scope is limited. It would benefit from a more diverse sampling strategy to capture a broader genetic diversity of BCoV and clarify the origins and spread of HE-insertion/deletion variants.

## Conclusion

These results suggest that BCoV HE deleted/inserted variants remain prevalent in cattle and exhibit different biological characteristics. These findings enhance our knowledge of the prevalence and evolution of BCoV. The current SARS-CoV-2 outbreak has again put coronavirus in the global spotlight [9]. We should be alert to these HE-variants with insertions or deletions in the RBD, which may increase the possibility of interspecies transmission.

## Materials and methods

### Clinical samples

From December 2021 to January 2022, feces and nasal swabs of 47 calves (< 2 months old) were collected from five farms in three cities of Ningxia Hui Autonomous Region, China, including one farm in Zhongwei City (36°06′N, 104°17′E), three farms in Wuzhong City (36°34′N, 105°17′E), and one farm in Yinchuan City (38°17′N, 105°50′E). These calves exhibited watery diarrhea, cough, and runny nose. All samples were transported to the laboratory using dry ice and stored at − 80 °C.

### RNA extraction and cDNA synthesis

The nasal swabs and fecal samples were re-suspended in phosphate-buffered saline (PBS) (1:5), centrifuged at 8,000 g for 15 min, and filtered through a 0.22-µm filter (Biosharp, China). According to the instructions, viral RNA was extracted from 400 µL of each sample suspension using *AG RNAex Pro* Reagent (Accurate Biology, China). cDNA was immediately synthesized using 5X *Evo* M-MLV RT Reaction Mix Ver.2 according to the manufacturer’s instructions (Accurate Biology, China) and stored at − 20 °C.

### BCoV detection and amplification of genes

Bovine coronavirus in feces and nasal swabs was identified using a PCR assay, as described previously (see additional file 6) [42]. Complete HE and S genes were amplified using PCR from BCoV-positive samples (see additional file 7). PCR was performed using Premix Taq™ (TaKaRa Taq ™ Version 2.0 plus dye) following the manufacturer’s instructions (see additional file 8). All PCR products were purified using the TIANquick Midi Purification Kit (TIANGEN, China) following the manufacturer’s instructions, after which they were ligated to the pMD19-T vector (TaKaRa Bio Inc., Japan) and transformed into DH5α competent cells for sequencing. Sequences were assembled using SeqMan software (version 7.0; DNASTAR Inc).

### Virus isolation and identification

We isolated BCoV as previously described [43]. The supernatants of 34 BCoV-positive samples were filtered with a 0.22-µm filter, inoculated into the HRT-18 cell monolayer (1: 10), and adsorbed at 37 ℃ for one hour. We discarded the supernatants and added 3 mL Dulbecco’s Modified Eagle Medium (DMEM) with 100 U/mL penicillin, 100 µg/mL streptomycin (Biosharp, China), 0.4 µg/mL TPCK trypsin (Sigma-Aldrich, St.Louis, MO, USA), and 2% fetal bovine serum (FBS) (ExCell, China). The cells were incubated at 37 °C and 5% CO_2_ for 96 h. The plaque technique purified the isolated strains once the CPE exceeded 80% after three generations of culture.

The presence of BCoV in a generation was detected by IFA, western blot, and electron microscopy, as previously described [44], and the presence of other major bovine diarrhea pathogens (bovine rotavirus and bovine viral diarrhea virus) was detected using RT-PCR [45] (see additional file 6). Virus titers (TCID_50_) were determined using IFA. Briefly, BCoV was serially diluted ten-fold in DMEM, then inoculated into HRT-18 cells pre-seeded in 96-well plates. After incubation for 60 min, cells were washed three times in PBS, and 100 µL DMEM medium containing 2% FBS was added to each well (eight replicate wells per dilution). The plates were incubated at 37 °C/5% CO_2_ for 72 h. The medium was discarded from the wells, and the plates were immunoreacted with an anti-BCoV nucleocapsid (N) polyclonal antibody (1:100) prepared in our laboratory for IFA. Viral titration was determined as TCID_50_/mL using the Reed-Muench method.

### Western blotting analysis

The HRT-18 cells pre-seeded in 24-well plates were infected with cultures identified as BCoV-positive by RT-PCR. Cells were collected 6, 12, 18, and 24 HPI. According to previously published protocols [46], cells were lysed using radioimmunoprecipitation assay buffer containing 1 mM phenylmethylsulfonyl fluoride on ice for 30 min and oscillated every ten minutes. Protein samples were separated by 12% SDS-PAGE and transferred onto PVDF membranes (Millipore, USA). The membranes were blocked with 5% skim milk and then incubated with indicated primary antibodies at 37 °C for two hours, followed by HRP-conjugated goat anti-mouse IgG (1:1000, CWBIO, China). Immunoreactive bands were visualized using an enhanced chemiluminescence western blotting analysis system (Thermo, USA). The density of immunoreactive bands was analyzed using Image J-v1.8.0. Primary antibodies were mouse anti-BCoV nucleocapsid (N) polyclonal antibody (1:20000, prepared in our laboratory) and mouse anti-glyceraldehyde-3-phosphate dehydrogenase (GAPDH) polyclonal antibody (1:20000, Proteintech Group, China).

### Genome sequencing of BCoV isolates

Four purified strains were selected for viral genome sequencing: the HE-insertion variant (B298-HE428), HE-deletion variant (B277b-HE420), HE non-variant (B277a-HE424), and enteric isolate (F226-HE424). Whole-genome sequencing was performed using next-generation sequencing at the Guangdong Magigene Biotechnology Co., Ltd. (Guangdong, China). Sequence data were analyzed using Blast (v2.9.0 +) software.

### Sequence, phylogeny, and recombination analysis

MEGA 7.0.26 was used to perform multiple sequence alignment using ClustalW and to build a phylogenetic tree. The phylogenetic tree based on the amino acid sequences was constructed using the maximum-likelihood method with bootstrap values derived from 1000 replicates and the Poisson model. The nucleotide sequence phylogenetic tree of the genome was similarly constructed using the maximum-likelihood method, with bootstrap values calculated from 1000 replicates and the Kimura 2-parameter model [47]. Meg Align software (DNASTAR, Inc.) was used for homology analysis of nucleotide and deduced aa sequences. Genome recombination analysis was performed using RDP (version 5.5) (seven methods: RDP, GeneConv, Chimaera, BootScan, SiScan, MaxChi, and 3Seq) and SimPlot software (version 3.5.1) as previously described [48, 49].

### Molecular docking

The SDF file of O-acetylated sialic (PubChem CID: 24801866) was obtained from the PubChem database (https://pubchem.ncbi.nlm.nih.gov) as a docking ligand. For the target protein, the crystal structure of bovine coronavirus hemagglutinin-esterase (SMTL ID: 3cl4.1) was selected as a reference [14], and SWISSMODEL (https://www.swissmodel.expasy.org/interactive/) was used to construct three-dimensional models of HE proteins with different variations. Use AutoDock Vina software (version 1.1.2) to prepare the ligands and proteins required for molecular docking. PyMOL software (version 4.3.0) (https://pymol.org/) was used to separate the original ligand and protein structure, dehydrate, and remove organic matter. AutodockTools (http://mgltools.scripps.edu/downloads) was used to hydrogenate, check the charge, specify the atom type as AD4 type, and construct a docking grid box for protein structure. After docking with Vina, the scores of protein-ligand docking combinations were calculated, and Pymol and Discovery Studio software were used for three-dimensional and two-dimensional force analysis and visualization.

### One-step growth curve

To determine the impact of these mutations on BCoV adaptation to HRT-18 cells, the HE-insertion variant (B298-HE428), HE-deletion variant (B277b-HE420), or HE non-variant (B277a-HE424) were inoculated into HRT-18 cells at the same multiplicity of infection (MOI = 1) for one hour at 37 °C. The cell cultures were harvested at 0, 12, 24, 36, 48, 60, 72, 84, 96, 108, and 120 HPI to analyze growth kinetics, as described [49]. After one freeze-thaw cycle, the supernatants were centrifuged to remove the cellular debris and stored at − 80 °C. TCID_50_ measured the viral infectivity according to the Reed and Müench method.

### Statistical analysis

GraphPad Prism (version 8.02) (GraphPad Software Inc., San Diego, CA, USA) software was used to analyze the data. The data were expressed as mean ± standard deviation and average values of three independent experiments were calculated. The differences in BCoV prevalence between different clinical samples were calculated using the χ^2^-test, and other statistical comparisons were using the Student’s *t*-test. Differences were considered significant by p-value (* *P* < 0.05; ** *P* < 0.01, *** *P* < 0.001, ns: not significant).

## Electronic supplementary material

Below is the link to the electronic supplementary material.

Supplementary Material 1

Supplementary Material 2

Supplementary Material 3: Additional file 3. Maximum-likelihood analysis in combination with 1,000 bootstrap replicates was used to derive a phylogenetic tree based on the complete E (a), M (b), and N (d) protein sequences. The sequences were aligned and clustered by Clustal W in MEGA 7.0 software

Supplementary Material 4: Additional file 4. The recombination analysis of 216 BCoV genome using SimPlot 3.5.1. The vertical axis indicates the similarity (%) of nucleotide sequences between the query strain and other reference strains. The horizontal axis indicates the nucleotide positions. SimPlot analysis was performed using a window size of 400 nt and step size of 200 nt

Supplementary Material 5: Additional file 5. Map of world showing the geographical distribution of the BCoV HE deleted/inserted variants collection sites. The HE-insertion variants were marked with a green triangle and the HE-deletion variants were marked with a blue circle

Supplementary Material 6

Supplementary Material 7

Supplementary Material 8

Supplementary Material 9: Uncropped full-length blots of BCoV-N and GAPDH protein. The black box is the cropped area

## Data Availability

The sequences reported in this study were submitted to GenBank under accession numbers OP762501-OP762511, OP762512-OP762519, and OP866726-OP866729.

## Abbreviations

BCoV: Bovine Coronavirus
HE: Hemagglutinin-Esterase
RBD: Receptor-Binding Domain
ORF1a: Replicase Polyproteins pp1a
ORF1b: Replicase Polyproteins pp1b
S: Spike
M: Membrane
MHV: Mouse Hepatitis Virus
E: Envelope
N: Nucleocapsid
HCoV-OC43: Human Coronavirus OC43
PBS: Phosphate-Buffered Saline
DMEM: Dulbecco’s Modified Eagle Medium
FBS: Fetal Bovine Serum
CPE: Cytopathic Effect
IFA: Indirect Immunofluorescence Assay
GAPDH: Glyceraldehyde-3-Phosphate Dehydrogenase
HPI: Hours Post-Infection
