# Supplementary figures and images for: Isolation and characterization of bovine coronavirus variants with mutations in the hemagglutinin-esterase gene in dairy calves in China
Source: BMC Vet Res. 2025 Feb 24;21:92. doi: 10.1186/s12917-025-04538-w (PMC11849235; doi:10.1186/s12917-025-04538-w)

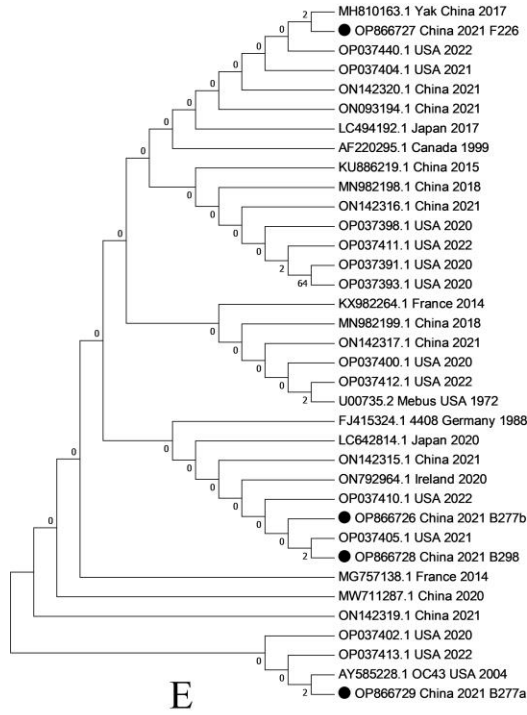

E

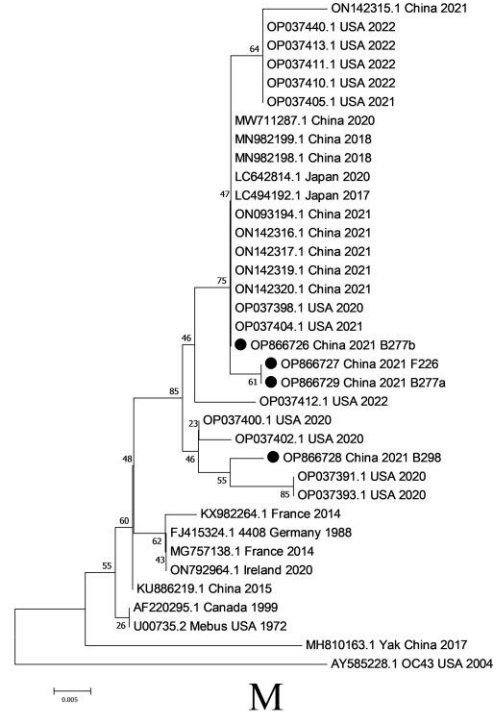

M

(a)

(b)

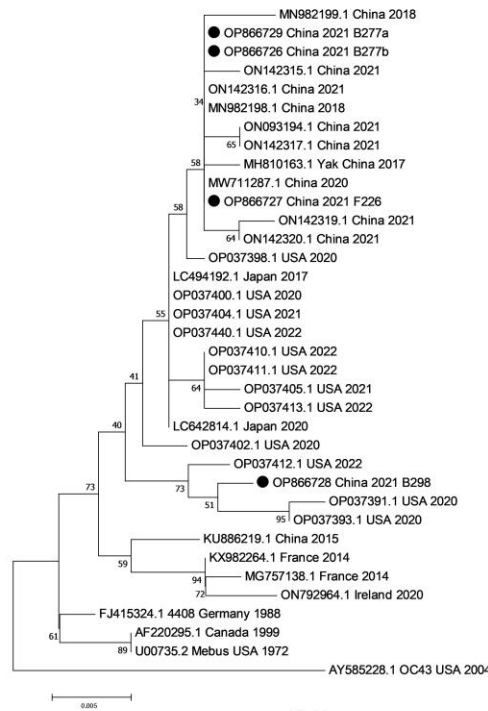

N

(c)

Supplement: Supplementary file 1 — Supplementary Material 1 [file 12917_2025_4538_MOESM1_ESM.pdf]

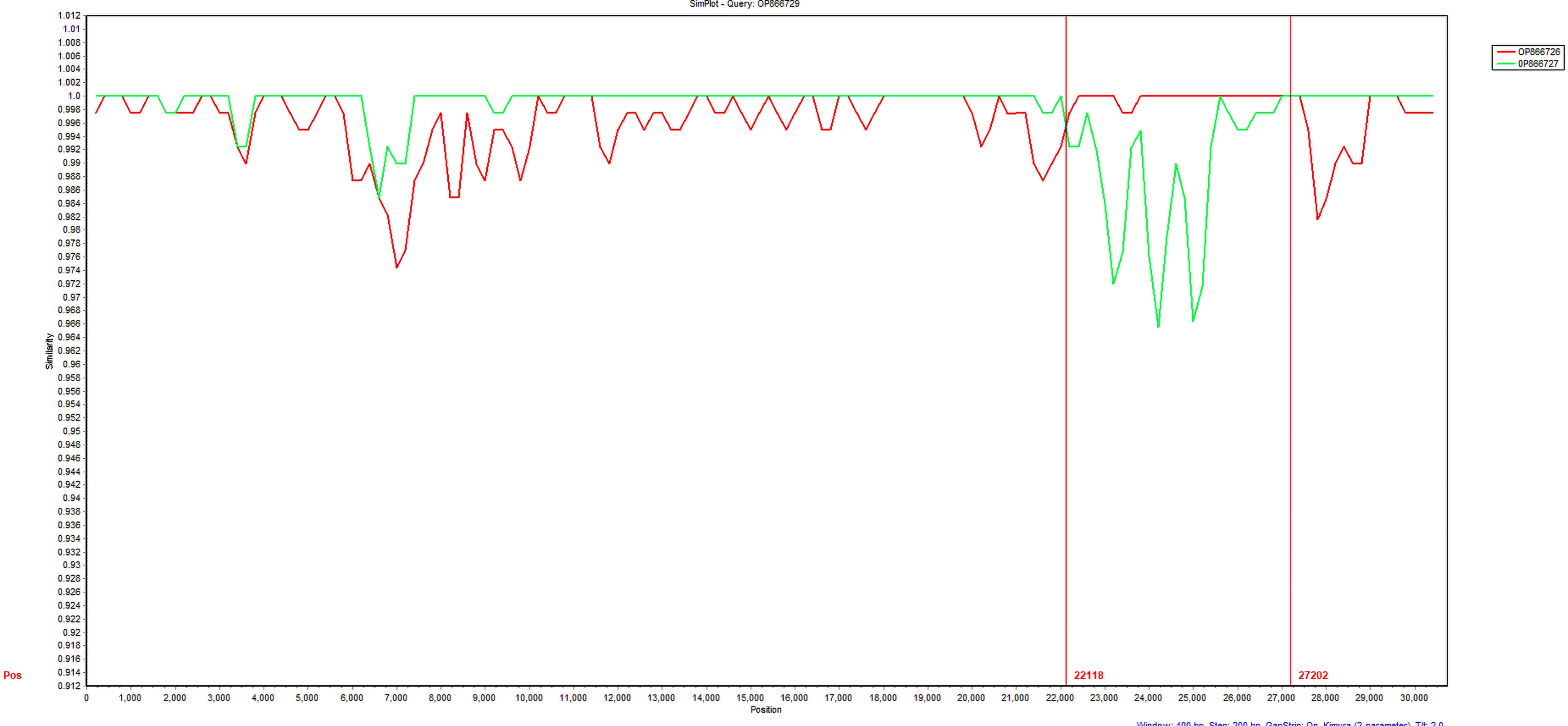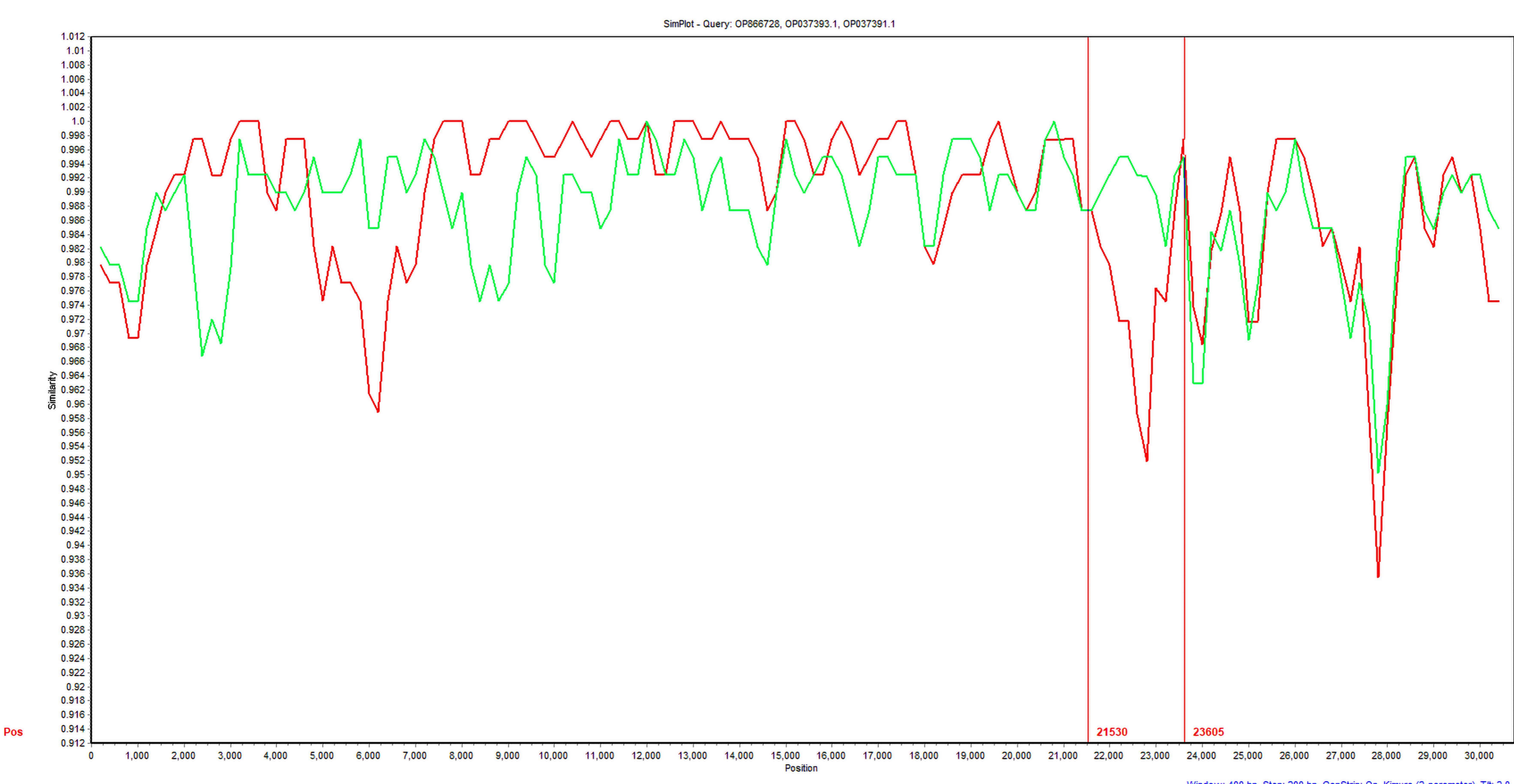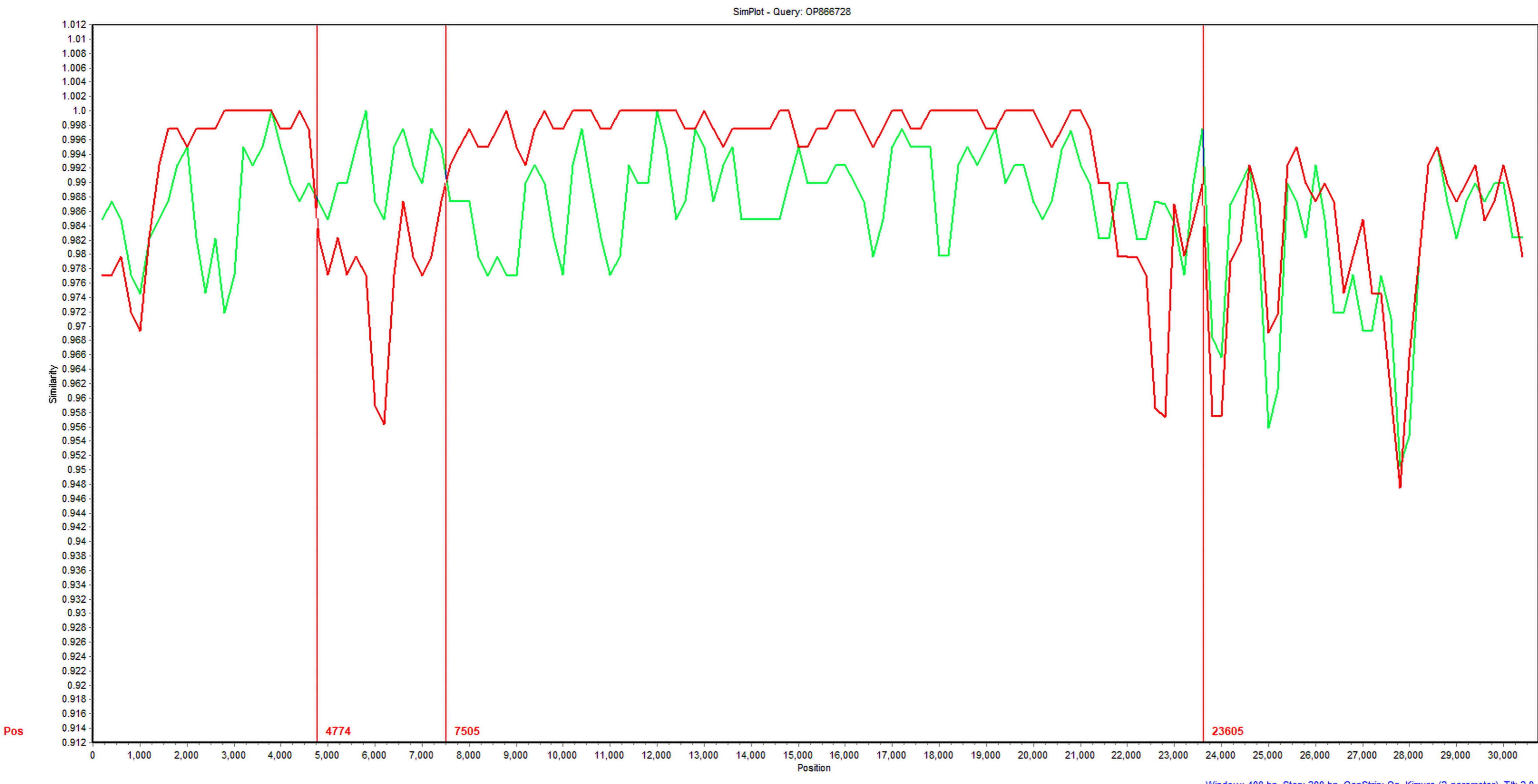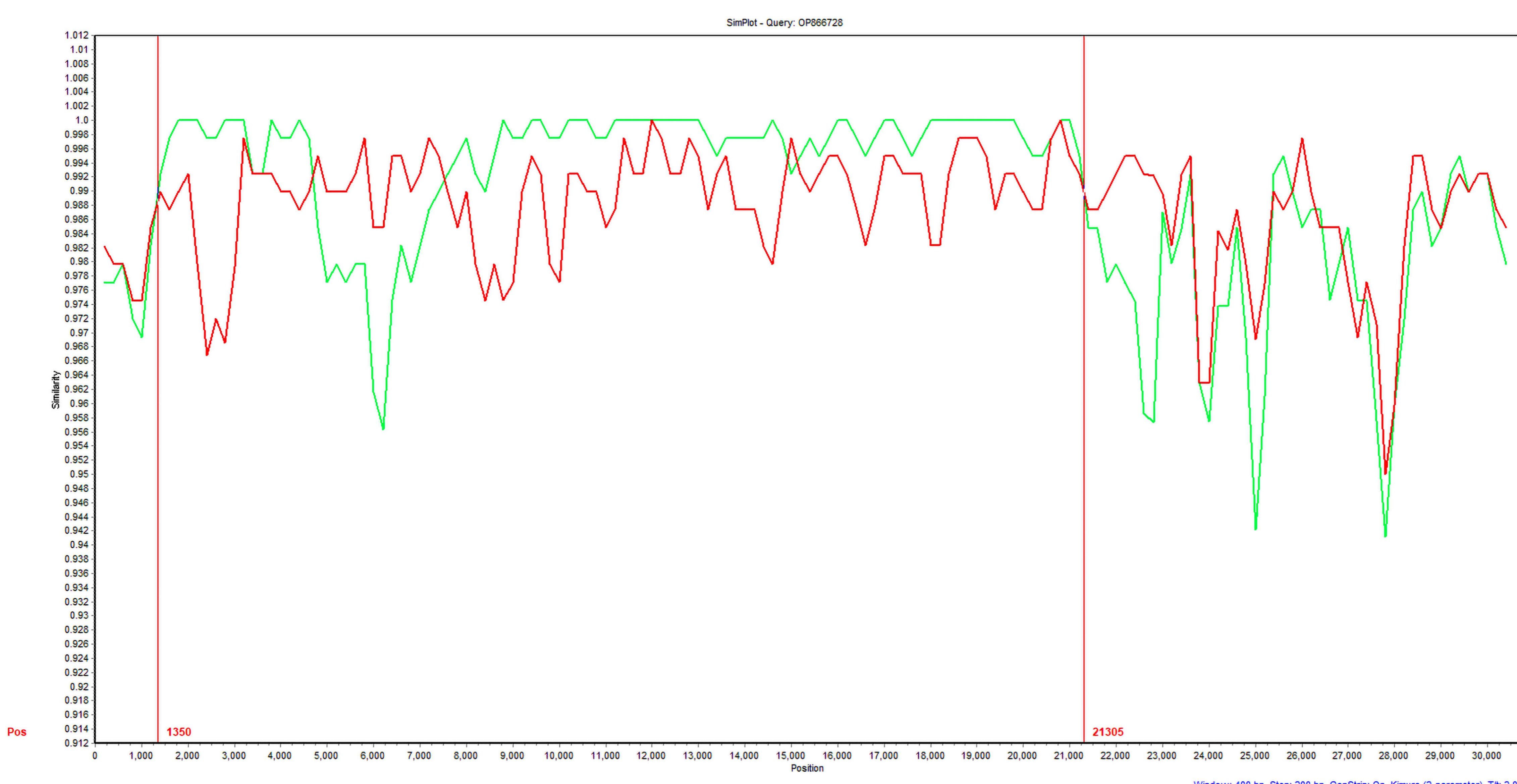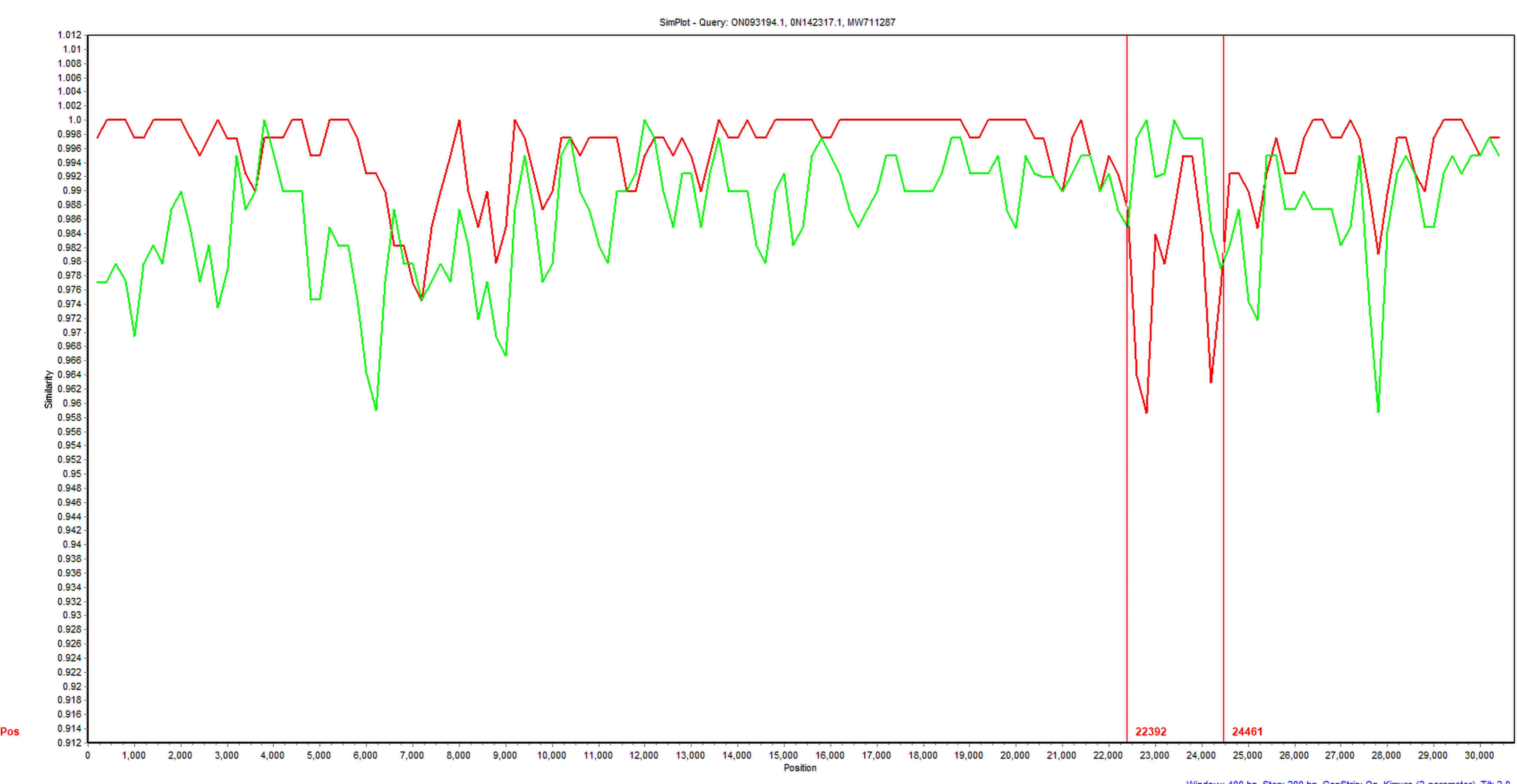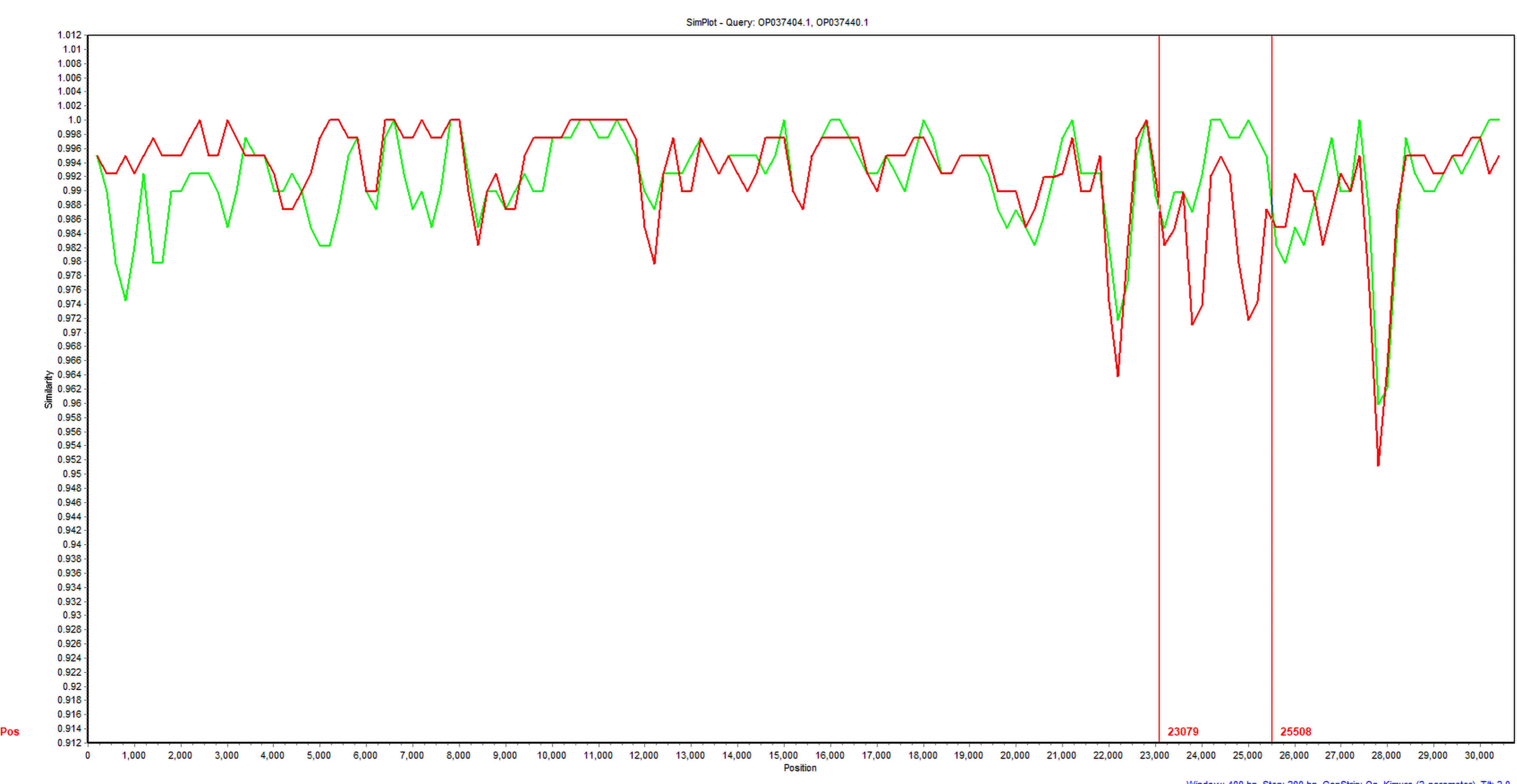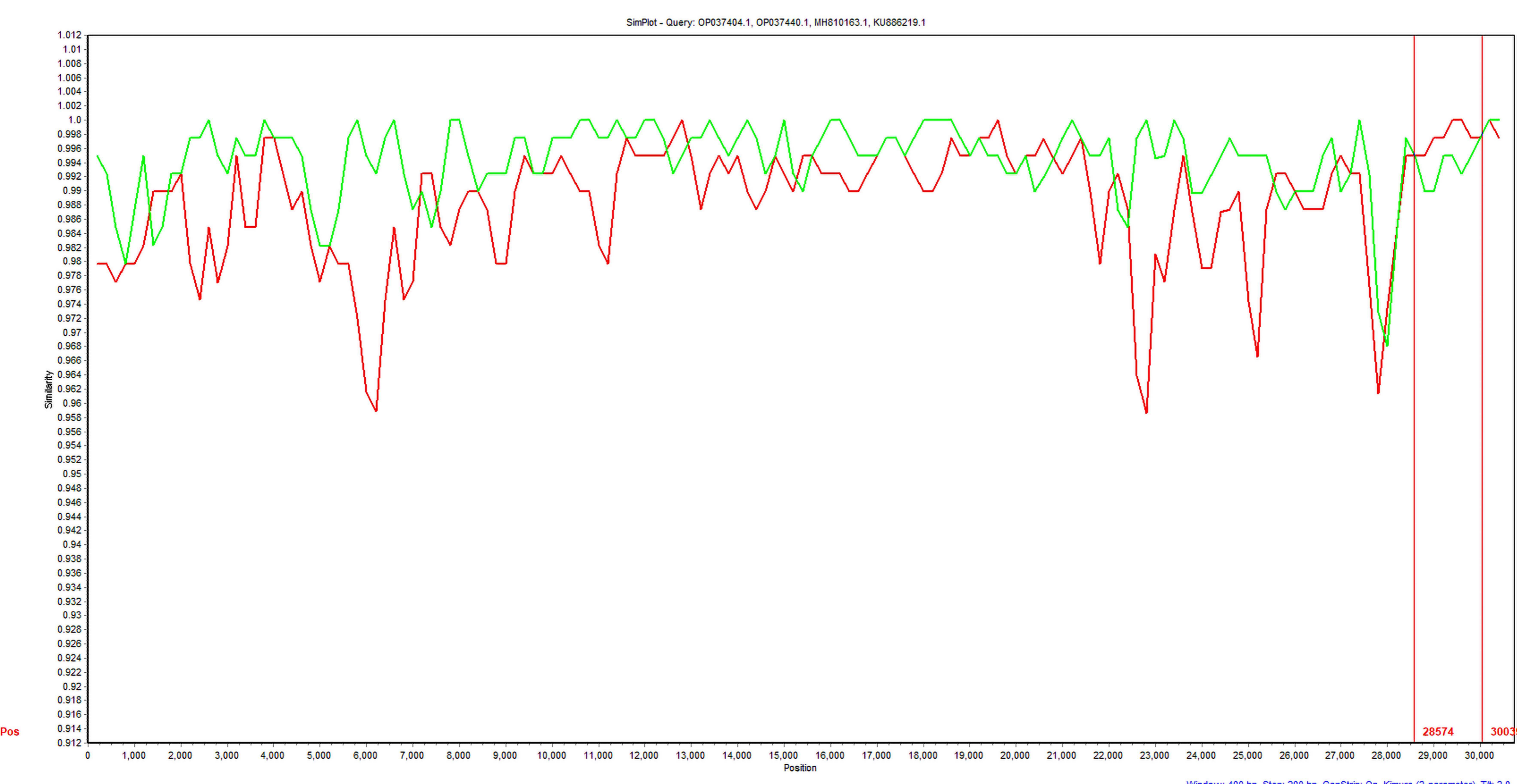

Supplement: Supplementary file 2 — Supplementary Material 2 [file 12917_2025_4538_MOESM2_ESM.pdf]

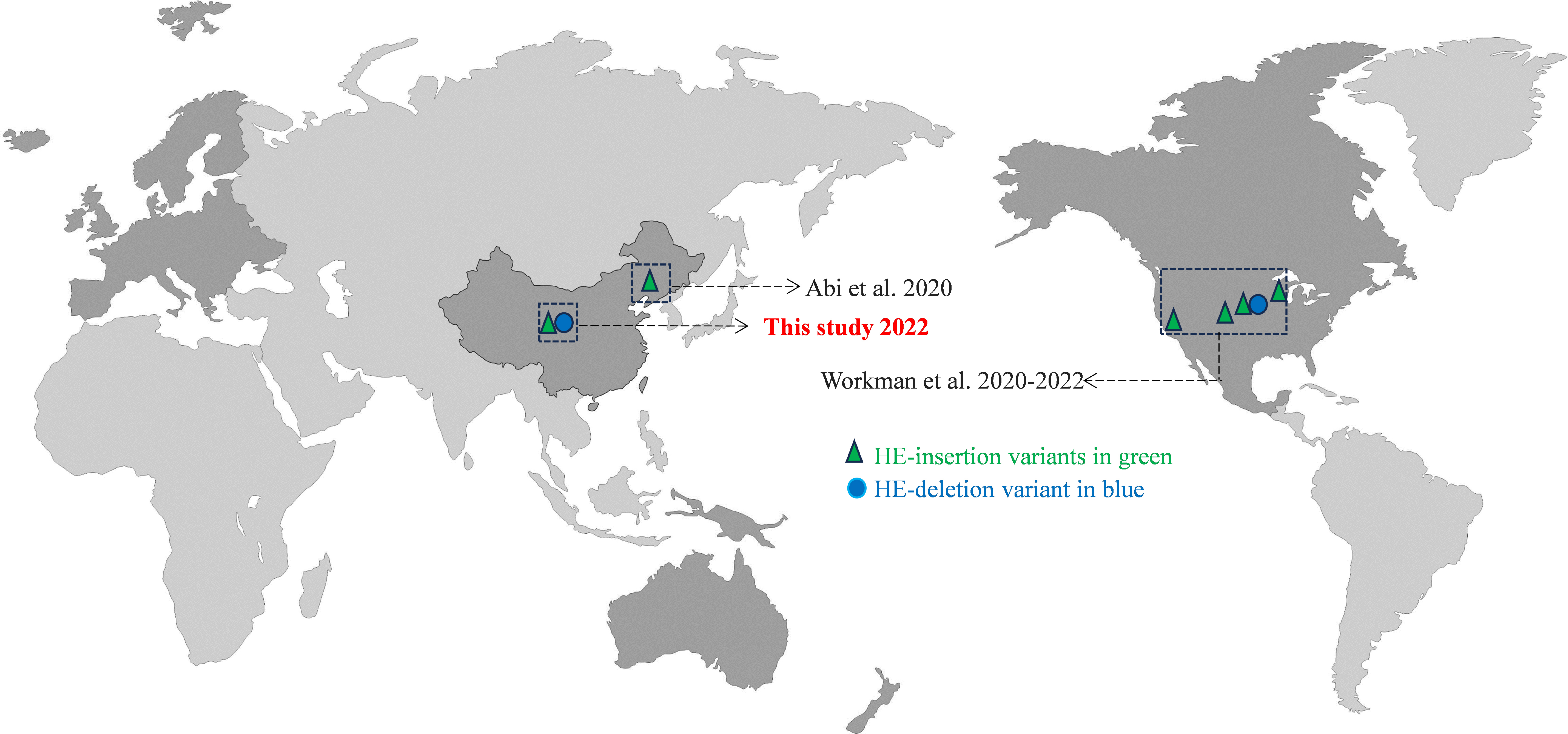

Supplement: Supplementary file 8 — Supplementary Material 8 [file 12917_2025_4538_MOESM8_ESM.pdf]

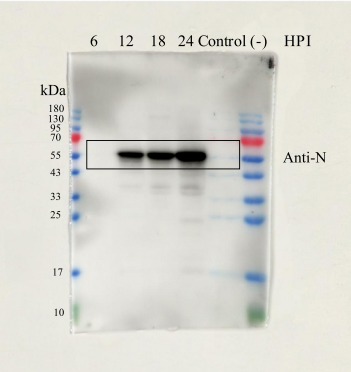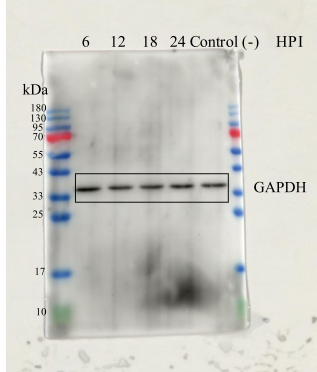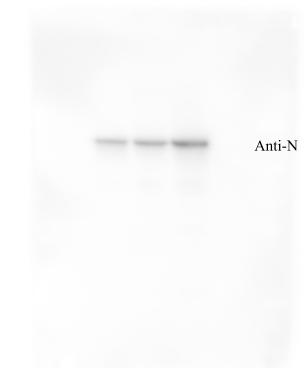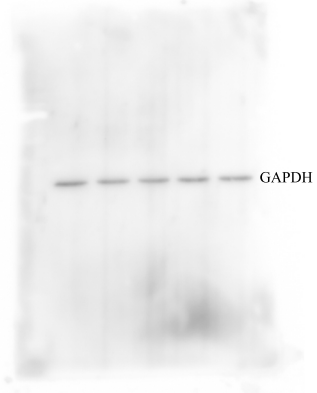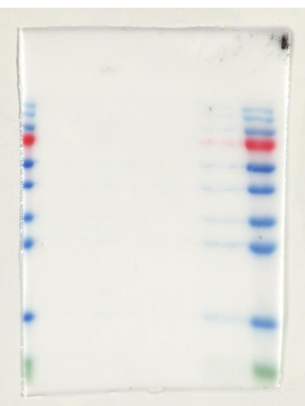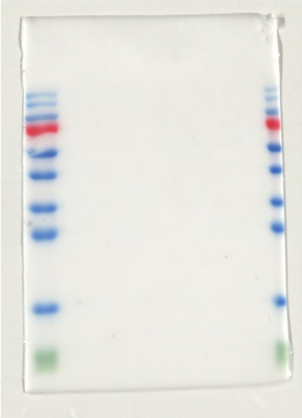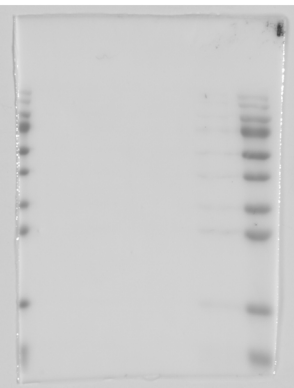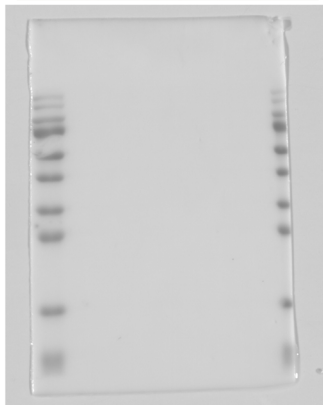

Supplement: Supplementary file 9 — Supplementary Material 9: Uncropped full-length blots of BCoV-N and GAPDH protein. The black box is the cropped area [file 12917_2025_4538_MOESM9_ESM.pdf]
