# Supplementary material for: Isolation and characterization of bovine coronavirus variants with mutations in the hemagglutinin-esterase gene in dairy calves in China
Source: BMC Vet Res. 2025 Feb 24;21:92. doi: 10.1186/s12917-025-04538-w (PMC11849235; doi:10.1186/s12917-025-04538-w)
Supplement: Supplementary file 3 — Supplementary Material 3: Additional file 3. Maximum-likelihood analysis in combination with 1,000 bootstrap replicates was used to derive a phylogenetic tree based on the complete E (a), M (b), and N (d) protein sequences. The sequences were aligned and clustered by Clustal W in MEGA 7.0 software [file 12917_2025_4538_MOESM3_ESM.pdf]

Table S1. The result of BCoV detection in diarrhoea faecal and nasal swab samples

Area	Number of farms	Health status	Fecal and swabs from the same individual	Numbers of samples		The positive rate %	
				faecal	swab	faecal	swab
Zhongwei	1	diarrhea and runny nose	Yes	24	24	12.5 (3/24)	50 (12/24)
Wuzhong	3	diarrhea and runny nose	Yes	16	16	37.5 (6/16)	62.5 (10/16)
Yinchuan	1	diarrhea and runny nose	Yes	7	7	14.29 (1/7)	28.57 (2/7)
Total	5	/		47	47	21.28 (10/47)	51.06 (24/47)
