# Supplementary material for: Isolation and characterization of bovine coronavirus variants with mutations in the hemagglutinin-esterase gene in dairy calves in China
Source: BMC Vet Res. 2025 Feb 24;21:92. doi: 10.1186/s12917-025-04538-w (PMC11849235; doi:10.1186/s12917-025-04538-w)
Supplement: Supplementary file 4 — Supplementary Material 4: Additional file 4. The recombination analysis of 216 BCoV genome using SimPlot 3.5.1. The vertical axis indicates the similarity (%) of nucleotide sequences between the query strain and other reference strains. The horizontal axis indicates the nucleotide positions. SimPlot analysis was performed using a window size of 400 nt and step size of 200 nt [file 12917_2025_4538_MOESM4_ESM.pdf]

Table S3. Primers of RT-PCR

Target gene	Primer name	Sequence (5′~3′)	length (bp)	Annealing temperature (℃)	Reference
BCoV-Nsp10 (detection)	BCoV-F	CGAGTTGAACACCCAGAT	230	49	[38]
	BCoV-R	CCAARTTYTAYGGHGGITGG			
	GAPDH-R	TGGAGGAGTGGGTGTCG			
BRV-VP6 (detection)	BRV-F	CCACCAGGTATGAATTGGAC	231	52	[38]
	BRV-R	CGCCATCTGAGTGATTACTC			
BVDV-5′ UTR (detection)	BVDV-F	GCCATGCCCTTAGTAGGACT	230	61	[41]
	BVDV-R	CTCTGCTGTACATGGCACAT			
