# Supplementary material for: Isolation and characterization of bovine coronavirus variants with mutations in the hemagglutinin-esterase gene in dairy calves in China
Source: BMC Vet Res. 2025 Feb 24;21:92. doi: 10.1186/s12917-025-04538-w (PMC11849235; doi:10.1186/s12917-025-04538-w)
Supplement: Supplementary file 5 — Supplementary Material 5: Additional file 5. Map of world showing the geographical distribution of the BCoV HE deleted/inserted variants collection sites. The HE-insertion variants were marked with a green triangle and the HE-deletion variants were marked with a blue circle [file 12917_2025_4538_MOESM5_ESM.pdf]

Table S5. PCR procedure for the complete S and HE gene amplification

Procedure	Temperature (℃)	Time	Cycle
Pre-denaturation	94	5 min	1
Denaturation	94	30 s	
Annealing	Optimum annealing temperature	30 s	35
Extension	72	1 min 30 s	
