# Supplementary material for: Isolation and characterization of bovine coronavirus variants with mutations in the hemagglutinin-esterase gene in dairy calves in China
Source: BMC Vet Res. 2025 Feb 24;21:92. doi: 10.1186/s12917-025-04538-w (PMC11849235; doi:10.1186/s12917-025-04538-w)
Supplement: Supplementary file 6 — Supplementary Material 6 [file 12917_2025_4538_MOESM6_ESM.pdf]

Table S2. Nucleotide or amino acid identities of strain B298-HE428 (OP866728.1) compared with other BCoV genome sequence

Strain	GenBank accession no.	Shared nucleotide identity (%)/amino acid identity (%)										
		Genome	ORF1a	ORF1b	NS2	HE	S	NS4.9	NS12.7	E	M	N
B277a-HE424	OP866729.1	98.9/-	99.2/99.2	99.8/99.9	98.3/98.2	96.0/97.4	98.1/98.0	92.2/83.3	97.0/93.6	99.6/100	98.6/97.8	98.7/98.7
B277b-HE420	OP866726.1	98.9/-	99.1/99.2	99.8/99.9	98.3/98.2	95.1/96.5	98.1/98.0	91.1/80.0	97.6/96.4	99.6/100	98.6/98.3	98.8/98.7
F226-HE424	OP866727.1	98.9/-	99.2/99.2	99.8/99.9	98.2/97.8	96.3/97.2	97.8/97.6	92.2/83.3	97.0/93.6	99.6/100	98.6/97.8	98.7/98.7
BCoV/A10ss	MN982199.1	98.7/-	99.0/99.0	99.4/99.9	98.6/98.6	96.8/98.8	98.1/97.9	91.1/83.3	97.6/95.5	99.6/100	98.7/98.3	98.4/98.2
BCoV/06/R	OP037402.1	98.6/-	98.7/99.0	99.1/99.6	99.0/99.3	96.7/97.7	98.2/98.1	96.7/90.0	97.6/95.5	99.6/100	99.0/98.7	98.9/98.9
BCoV/03/R	OP037412.1	97.9/-	98.6/98.9	98.9/99.7	98.3/98.9	98.1/99.1	98.0/97.9	98.9/96.7	97.6/95.5	99.6/100	98.4/97.8	99.4/99.3
BCoV/Mebus	U00735.2	98.1/-	98.5/98.6	98.7/99.4	97.0/96.4	96.4/97.7	97.0/95.8	91.1/76.7	97.6/96.4	98.8/98.8	98.4/97.8	97.8/98.2
