# Supplementary material for: Isolation and characterization of bovine coronavirus variants with mutations in the hemagglutinin-esterase gene in dairy calves in China
Source: BMC Vet Res. 2025 Feb 24;21:92. doi: 10.1186/s12917-025-04538-w (PMC11849235; doi:10.1186/s12917-025-04538-w)
Supplement: Supplementary file 7 — Supplementary Material 7 [file 12917_2025_4538_MOESM7_ESM.pdf]

Table S4. Primers used for amplifying the full-length HE and S gene

Target gene	Primer name	Sequence (5′~3′)	length (bp)	Reference
HE	893F	GTTATTGTTGTTTAGAAGTGGTGCGT	893	This study
	893R	ATTGACTATCATCATAATACTTTGTA		
	1144F	CTGTGTATCGCAGCCTTACTTTTGTT	1144	
	1144R	CAGTGTGCTCAATAGTAAAGTTCCCT		
S	1263F	ATAGGAGATTTAAAGTGTACTACAGT	1263	
	1263R	TACAACTGACAACTTGTAGCAGTAGT		
	1387F	TTTATTCAGGCAGACTCATTTACTTG	1387	
	1387R	CACAGACAAAAGCAGAACAATCAATA		
	1255F	TTACTGTGTGGATTACTCTACAAAAA	1255	
	1255R	CGGGACTAACCTTCGCAGTGACATAC		
	798F	TACCGCTCTTAATGCTTATGTTTCTC	798	
	798R	TGTGATGTTTTAATTACTAACTCCTG		
	524F	GAACATTTCAACACCCAACCTCCCTG	524	
	524R	CAGTACCCACTAAACAGCAGGCATTG		
